# AmpWrap: a one-line fully automated amplicon metabarcoding 16S and 18S rRNA gene analysis

**DOI:** 10.1093/bioadv/vbaf312

**Published:** 2025-12-02

**Authors:** Lapo Doni, Alessia Marotta, Luigi Vezzulli, Emanuele Bosi

**Affiliations:** Department of Earth, Environmental and Life Sciences (DISTAV), University of Genoa, 16132 Genoa, Italy; National Biodiversity Future Center (NBFC), 90133 Palermo, Italy; Department of Earth, Environmental and Life Sciences (DISTAV), University of Genoa, 16132 Genoa, Italy; Department of Earth, Environmental and Life Sciences (DISTAV), University of Genoa, 16132 Genoa, Italy; National Biodiversity Future Center (NBFC), 90133 Palermo, Italy; Department of Earth, Environmental and Life Sciences (DISTAV), University of Genoa, 16132 Genoa, Italy; National Biodiversity Future Center (NBFC), 90133 Palermo, Italy

## Abstract

**Motivation:**

The revolution of next-generation sequencing has driven the establishment of metabarcoding as an efficient and cost-effective method for exploring community composition. Amplicon sequencing of taxonomic marker genes, such as the 16S rRNA gene in prokaryotes, provides an efficient method for high-throughput taxonomic profiling. The advent of long read technologies made it feasible to sequence the whole 16S rRNA gene rather than only a few regions, with the potential to achieve species-level resolution. Despite the affordability and scalability of such experiments, a major bottleneck remains the lack of integrated and user-friendly analytical workflows. Current pipelines often require the use of multiple tools with complex dependencies, and parameter optimization is frequently performed manually, limiting reproducibility and overall efficiency.

**Results:**

To address these limitations, we developed, AmpWrap, an automated, one line workflow designed to analyse both Illumina and Nanopore amplicons, requiring minimal efforts by the user and automatically optimizing the trimming parameter to retain the maximum number of reads and information while reducing noise.

**Availability and implementation:**

AmpWrap is available at: https://github.com/LDoni/AmpWrap

## 1 Introduction

Since the pioneering studies on the 16S rRNA gene in 1977 by Carl Woese, this gene has been the gold-standard taxonomic marker for prokaryotic microorganisms (Santos *et al.* 2020). The characterization of prokaryotic communities through 16S rRNA gene sequencing has become increasingly more affordable due to the advent of high-throughput sequencing technologies (Buchner *et al.* 2022), and the continuous reduction of associated costs, turning this method into one of the most widely used among the scientific community (Santos *et al.* 2020). Nowadays, metabarcoding analysis is highly standardized due to the presence of established reference databases and pipelines, i.e. DADA2 (Callahan *et al.* 2016) and QIIME2 (Bolyen *et al.* 2019) for short reads, and EMU for long reads (Curry *et al.* 2022). Typically, all pipelines implement similar analytical steps to produce three main output files, which are used in the subsequent biostatistical analyses: the representative sequences, the frequency, and taxonomy tables. A current limitation of these approaches is that their implementations are not fully automated from raw data to these outputs, requiring users to define key parameters at different analytical steps, including quality filtering, clustering thresholds, and taxonomic assignment confidence levels, all of which can significantly impact the results. This is especially true for DADA2, which resolves amplicon sequence variants (ASVs) distinguishing sequence variants that differ by one nucleotide. Therefore, trimming parameters must be accurately set to maximize post-trimming sequence retention and information while minimizing expected sequencing errors. To this end, we developed AmpWrap, a fully automated, one-line command workflow for 16S and 18S rRNA gene metabarcoding analysis. It comprises two distinct modules, one for short reads (i.e. Illumina) and the other for long reads (i.e. Nanopore). All parameters, including DADA2 trimming parameters, are automatically determined, allowing to obtain with minimal efforts report-ready results from raw reads with the highest accuracy.

## 2 AmpWrap

**Figure 1.**
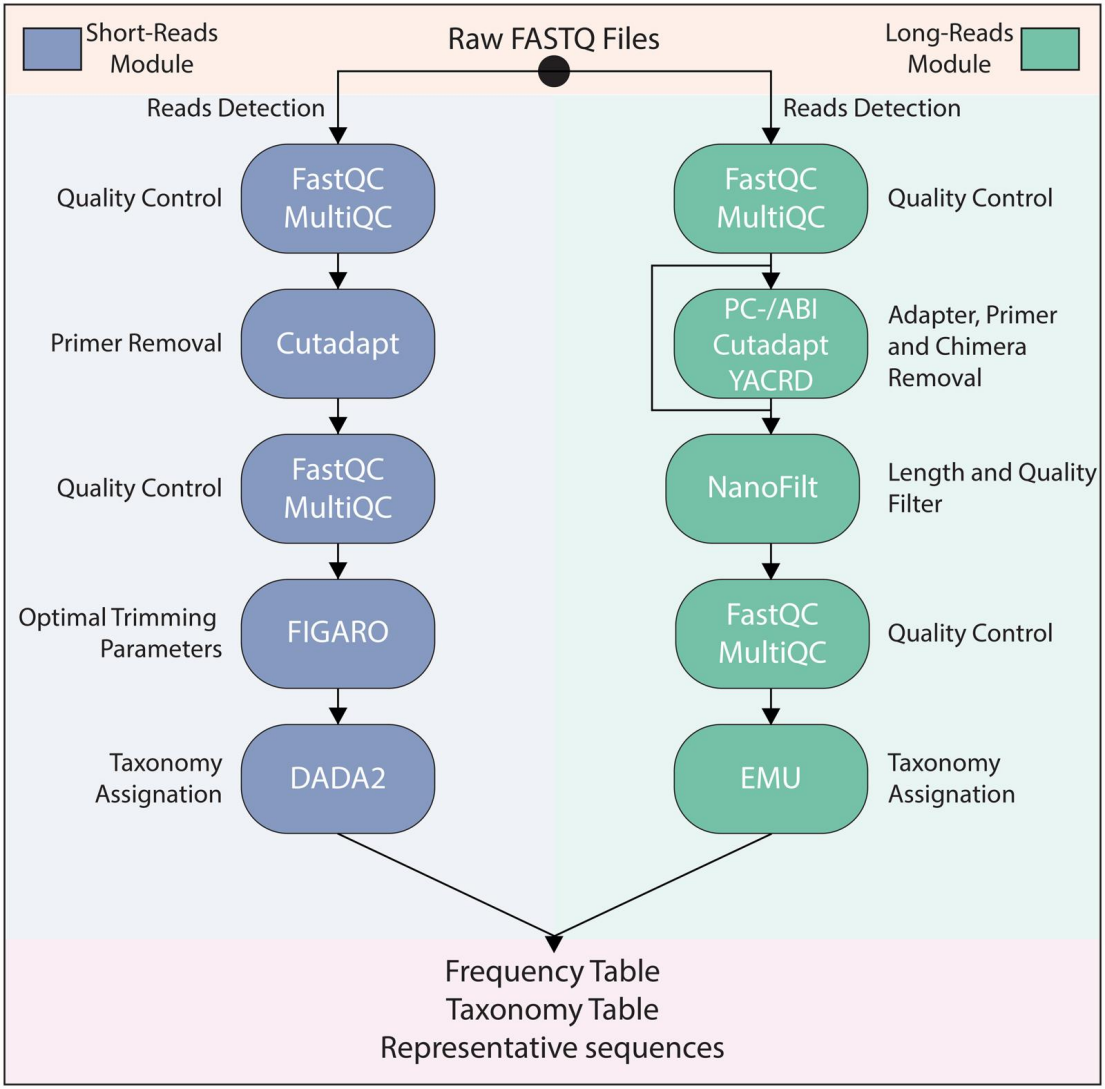
Schematic flowchart of the AmpWrap workflow, highlighting tools, inputs, and outputs. The short reads module involves quality control and adapter trimming using FastQC, MultiQC, and Cutadapt. A second quality control step is then performed. FIGARO analyses the raw reads to determine optimal trimming parameters for DADA2. Clean reads are then used as input for DADA2, where they undergo denoising, merging, chimaera removal, and ASV inference using the parameters generated by FIGARO. The long reads module includes quality control with FastQC and MultiQC. The user can either trim the adapters (PC-/ABI: Porechop or Porechop-ABI), primers (Cutadapt) and remove chimaeras (YACRD) or skip these steps. Quality and length filtering are then applied using NanoFilt, followed by a second quality control step. EMU processes the clean reads to generate the final outputs. Both modules produce a frequency table, a taxonomy table and representative sequences.

AmpWrap is designed as a reproducible, automated preprocessing workflow for amplicon sequencing data. Unlike other workflows, AmpWrap implements the automation of trimming parameters for DADA2 denoising using FIGARO, enabling users to really complete the preprocessing workflow with one-line analysis for both 16S and 18S rRNA genes. AmpWrap consists of two modules capable of analysing Illumina and Nanopore amplicon data (Fig. 1) and does not perform biostatistical analysis, which is handled by other pipelines (Table 1).

**Table 1.** Comparison between AmpWrap and other widely used workflows for 16S rRNA gene metabarcoding analysis (Callahan *et al.* 2016, Bolyen *et al.* 2019, Straub *et al.* 2020, Weißbecker *et al.* 2020, Ansorge *et al.* 2021).

Feature	AmpWrap	Dadaist2	DadaSnake	QIIME2	nf-core/ampliseq	DADA2 R package
Easy to run	✔	✗	✗	✗	✗	✗
Automation of trimming parameters	✔	✗	✗	✗	✗	✗
One-line analysis	✔	✔	✔	✗	✔	✗
Illumina short-read	✔	✔	✔	✔	✔	✔
Nanopore long-read support	✔	✗	✗	✗	✗	✗
Complete pre-processing workflow	✔	✗	✗	✗	✗	✗
Biostatistacal data analysis	✗	✔	✔	✔	✔	✗
Alternative short reads denoisers to DADA2	✗	✗	✔	✔	✗	✗
Complete summary report	✔	✔	✗	✗	✔	✗
Automatic sample detection	✔	✔	✗	✔	✔	✔
Multi marker support	✔	✔	✔	✔	✔	✔
Batch-merging multiple runs	✔	✔	✔	✔	✔	✔
Hand-off BIOM	✔	✔	✔	✗	✔	✗
Hand-off phyloseq	✔	✔	✔	✗	✔	✔

Batch-merging of multiple runs is supported, and comprehensive reports are generated on preprocessing metrics. We encourage users to include these reports directly as supplementary information for the preprocessing steps to ensure transparency and reproducibility in their manuscripts. AmpWrap is ideal for users who require an easy-to-run, automated, and reproducible preprocessing workflow that maximizes read retention and ensures transparent reporting. AmpWrap is available through the GitHub repository (https://github.com/LDoni/AmpWrap), while dependencies can be installed with Mamba or Conda ensuring quick and easy installation. Moreover, a Dockerfile and a pre-built Docker image that can be pulled directly from GitHub Container Registry for one-line installation across HPC/workstations. A frozen conda lockfile ensures reproducibility. Versioned releases are published on GitHub, and the repository is linked with Zenodo to provide persistent identifiers for each release.

The GitHub repository includes a comprehensive tutorial covering the setup process, basic and advanced usage of the pipeline, mock community testing examples, and real-world testing examples of 16S and 18S rRNA gene amplicons. The software has separate modules for short Illumina and long Nanopore reads (Fig. 1). To run a complete analysis for short reads, the required inputs are the path to the folder containing the raw reads, the sequences of the primers used for amplification and the amplicon size. Ampwrap short module detects paired-end fastq data *(.fq, .fq.gz, .fastq*, and *.fastq.gz*) in the specified folder with one of two primary file naming formats.

The Illumina Standard Format: <group>_<sample>_S##_L###_R[12]001.[fastq|fq][.gz] (i.e. ProjectA_Sample1_S1_L001_R1_001.fastq.gz; ProjectA_Sample1_S1_L001_R2_001.fastq.gz). The second accepted format is a Custom format, which provides a more flexible and generic naming scheme: <sample>[Rr][12].[fastq|fq][.gz] (i.e. SampleA_R1.fastq.gz; SampleA_R2.fastq.gz or S1_r1.fq.gz; S1_r2.fq.gz). User-supplied options are used to generate a Snakemake configuration file, then the Snakemake workflow automatizes all the steps of the pipeline ensuring optimal use of computational resources. The pipeline includes an initial quality control step using FastQC (Andrews 2010) and MultiQC (Ewels *et al.* 2016), followed by primer removal with Cutadapt (Martin 2011). Quality control is repeated after primer removal. Optimal parameters for DADA2 denoising are automatically determined with FIGARO (Weinstein *et al.* 2019), after which ASVs table and representative sequences files are generated with DADA2. Using Illumina primers CCTACGGGNGGCWGCAG (forward) and GACTACHVGGGTATCTAATCC (reverse), the V3–V4 amplicon typically displays two fragments ∼440–460 nt in length. Incorrectly setting the amplicon length parameter (−l) in ampwrap can lead to suboptimal analysis in DADA2, potentially impacting downstream noise reduction and taxonomic assignment. Therefore, for the calculation of the FIGARO parameters, for amplicons with some expected biological variation in length, it is best to use the longest expected size. Users can select among multiple methods and reference databases for taxonomic assignment. For 16S rRNA gene data, options include DECIPHER (Murali *et al.* 2018) with GTDB r226, SILVA SSU r138.2, or RDP v18 Silva 138, as well as the DADA2 built-in naive Bayesian classifier, which supports RDP v19, SILVA v138.2, and RefSeq + RDP v16 reference databases. For the 18S rRNA gene amplicon, users can choose between the PR2 v 5.1.1 and 18S Silva v132 reference databases. For the long reads module (Nanopore), the only required input is the path to the folder containing the raw reads. AmpWrap will detect these files, build the configuration file and launch the Snakemake workflow. Quality control is performed with FastQC and MultiQC, adapter trimming is optional and can be performed with either Porechop (Wick *et al.* 2017) or Porechop_ABI (Bonenfant *et al.* 2022). Also, primer trimming can be performed with Cutadapt (Martin 2011) and optionally chimaeras can be removed with YACRD (https://github.com/natir/yacrd). NanoFilt is used to filter reads based on length and quality. Subsequently, EMU (Curry *et al.* 2022) is used to infer the species-level taxonomic abundance, supporting its built-in database and both the Silva v138 and RDP v11.5 databases for 16S rRNA gene amplicons and PR2 v 5.1.1 database for 18S rRNA gene amplicons. EMU can provide species-level assignments, but some bacteria share identical or nearly identical 16S rRNA gene sequences. This may impact the analysis, so clinical or critical identifications should be confirmed with complementary loci or whole-genome approaches.

All critical information of the analysis (such as primers, read counts throughout different steps, the used databases, etc.) are included in a final report, aiming to facilitate the dissemination of the workflow results and to increase the reproducibility.

The three main output files (i.e. the representative sequences, the frequency, and taxonomy tables), generated by AmpWrap can be further processed with other widely used tools, such as MicrobiomeAnalyst (Lu *et al.* 2023), TaxonTableTools (Macher *et al.* 2021), or Phyloseq (McMurdie and Holmes 2013), for the biostatistical analyses. Moreover, a Phyloseq object and a .BIOM file are automatically generated and stored in the results folder. AmpWrap was developed and tested in Linux environments with different distributions (Linux Mint 20, Ubuntu 24.04.1 LTS, and WLS with Ubuntu 24.04.2 LTS). The pipeline automatically manages and installs the following verified versions within its environment: R v4.3.1, EMU v3.5.1, Snakemake v7.32.4, and Python v3.9.19.

By automating and integrating all analytical steps into a single command-line execution, AmpWrap minimizes manual operations, accelerates processing, standardizes workflows, and reduces the chance of human error. This significantly enhances reproducibility, making it a powerful tool for 16S and 18S rRNA gene metabarcoding studies.

## 3 Evaluation using mock community standards

Mock samples produced with Illumina Miseq and Nanopore sequencing (Stevens *et al.* 2023) were used to evaluate the workflow. Metabarcoding data was generated using the ZymoBIOMICS mock community standard, consisting of Gram-negative and Gram-positive bacteria and yeast with varying sizes and cell wall composition. For Illumina two samples were analysed (SRR20752610 and SRR20752596), the 341F/806R primers were used to amplify the V3-V4 regions of the 16S rRNA gene, while for Nanopore sequencing one sample (SRR20752542) was analysed and the 27F/1492R primers were used for the amplification. Results obtained using AmpWrap with the Silva database for both short and long reads (with emu—min-abundance 0.01 to avoid false positives) showed the recovery of all eight bacterial genera expected in the community (Fig. 1, available as supplementary data at *Bioinformatics Advances* online). Spearman correlations revealed a strong (*r *> 0.9) and significant (*P *< .05) association between the compositional abundance obtained and the theoretical ZymoBIOMICS values.

## Supplementary Material

vbaf312_Supplementary_Data

## Data Availability

The data underlying this article are available in *AmpWrap* at https://github.com/LDoni/AmpWrap and can be accessed with *10.5281/zenodo.17513793*.
