# Supplementary figures and images for: AmpWrap: a one-line fully automated amplicon metabarcoding 16S and 18S rRNA gene analysis
Source: Bioinform Adv. 2025 Dec 2;5(1):vbaf312. doi: 10.1093/bioadv/vbaf312 (PMC12701800; doi:10.1093/bioadv/vbaf312)

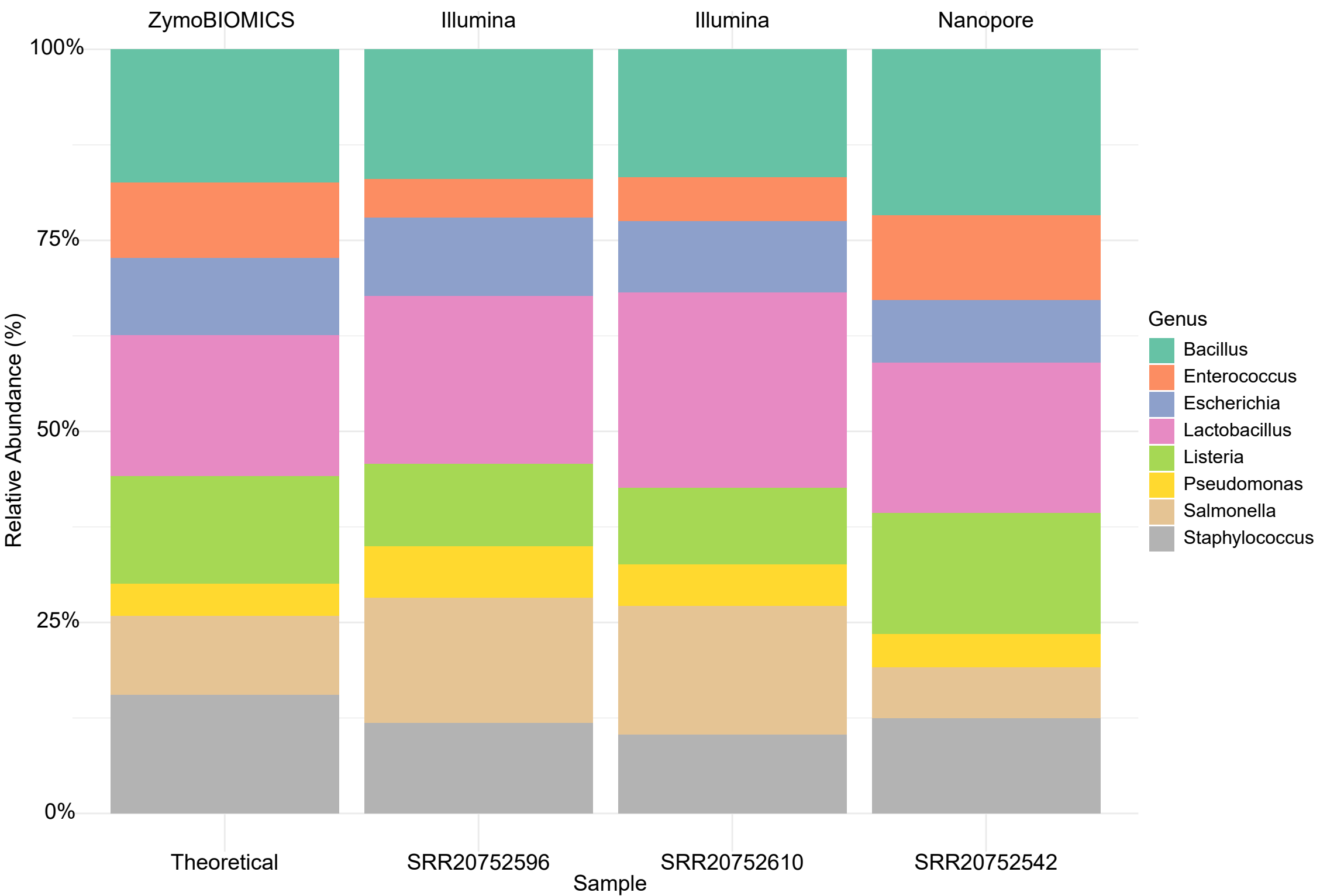

Supplement: vbaf312_Supplementary_Data [file vbaf312_supplementary_data.pdf]
